# Autoimmune Progesterone Dermatitis: A Case Report and Review of Literature

**DOI:** 10.7759/cureus.106760

**Published:** 2026-04-09

**Authors:** Maria AlSulami, Jana K Alharbi, Atheer Alzahrani, Manar Alghamdi, Khalid Al Hawsawi

**Affiliations:** 1 Department of Medicine and Surgery, College of Medicine, Umm Al-Qura University, Makkah, SAU; 2 Department of Dermatology, King Fahad Armed Forces Hospital, Jeddah, SAU; 3 Department of Dermatology, King Abdulaziz Hospital, Makkah, SAU

**Keywords:** autoimmune progesterone dermatitis, hypersensitivity reaction, progesterone, skin lesions, urticaria

## Abstract

Autoimmune progesterone dermatitis (APD) is a rare hypersensitivity reaction triggered by progesterone, predominantly affecting women in the luteal phase of the menstrual cycle. This case describes a 50-year-old woman with hypothyroidism and prolonged use of oral contraceptives, who presented with recurrent pruritic skin lesions occurring 3-10 days before menstruation and resolving post-menses. Clinical examination identified the lesions as urticarial plaques. After ruling out other conditions, such as urticarial vasculitis and autoinflammatory syndromes, a diagnosis of APD was established. Despite normal skin biopsy and blood tests, the patient responded well to symptomatic treatment, including antihistamines and topical corticosteroids. Treatment strategies were centered on ovulation suppression with oral contraceptives, while considering anti-progesterone therapies. The patient was reassured and placed on follow-up. While rare, APD should be considered in women with cyclical skin reactions, and diagnosis is primarily clinical, supported by intradermal progesterone testing and response to treatment.

## Introduction

Autoimmune progesterone dermatitis (APD) is a rare skin reaction to progesterone, predominantly affecting women of reproductive age, with higher prevalence in the third decade of life. The exact pathogenesis of APD remains unclear, though it is believed that prior exposure to exogenous progesterone may sensitize certain individuals, triggering an immune response upon subsequent exposure [1]. Symptoms typically manifest during the luteal phase, 3 to 10 days before menstruation, and subside shortly after menstruation begins [1]. Clinical presentations include eczema, erythema multiforme-like lesions, fixed drug eruption-like rashes, folliculitis, stomatitis, vesiculobullous eruptions, and urticaria [2]. Diagnosis is based on the correlation of symptoms with the menstrual cycle, confirmed by a skin or systemic reaction to an intradermal progesterone (IDP) injection [1].

Understanding the etiology of autoimmune progesterone dermatitis and its association with the menstrual cycle is essential for accurate diagnosis, appropriate management, and improved patient outcomes [2].

The prevalence of APD is unknown, with fewer than 90 cases reported in the English literature [2-4]. This report presents a case of APD and reviews its clinical features, diagnostic approaches, and treatment options.

## Case presentation

**Figure 1:**
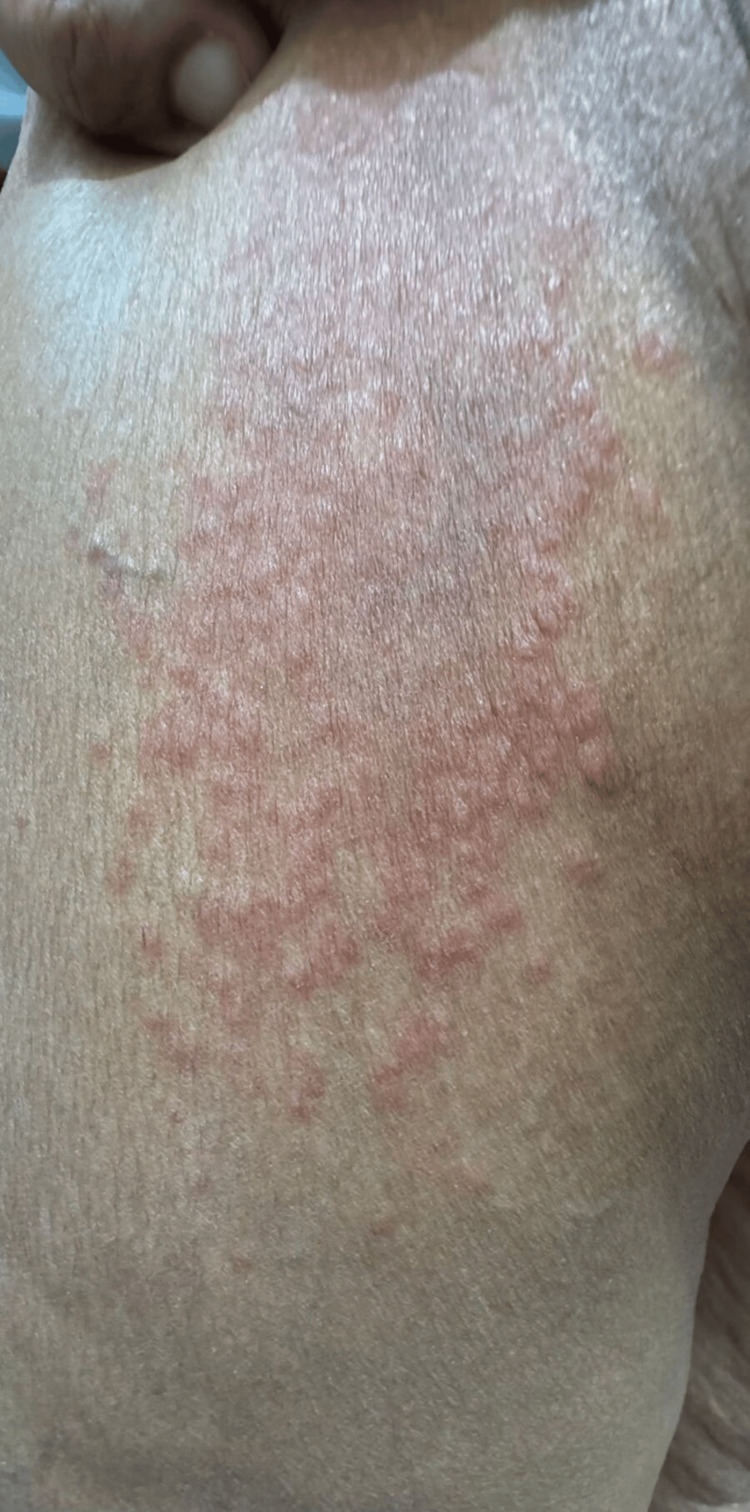
Well-demarcated, multiple, grouped, erythematous, urticarial papules and plaques with cyclical recurrence affecting the patient’s left thigh

A 50-year-old female, known case of hypothyroidism on levothyroxine and on oral contraceptive pills (OCP) for over 10 years, presented with a history of periodic, very pruritic skin lesions for three years. The skin lesions occurred a week prior to her menses and resolved completely a few days after menses, without leaving any sequelae. The itching improved with 0.1% betamethasonevalerate ointment and cetirizine 10 mg tab once daily, but the skin lesions were persistent until a few days post menses. The skin lesions were not associated with any prodromal symptoms like fever, joint pain, or malaise. General and systemic examinations were within normal limits, and past medical history and family history were unremarkable apart from what is mentioned above. Cutaneous examination revealed multiple urticarial plaques with dusky centers, measuring about 5-10 x 7-15 cm on her back, arms, legs, and thighs bilaterally (Figure 1).

The differential diagnosis included urticarial vasculitis, Schnitzler syndrome, systemic-onset idiopathic arthritis (adult-onset Still disease), familial Mediterranean fever (FMF), Muckle-Wells syndrome (MWS), and familial cold autoinflammatory syndrome (FCAS).

A skin biopsy was done, which showed no significant epidermal or dermal abnormalities, with no evidence of dermal edema or eosinophilic infiltration. Blood work is shown in Table 1. 

**Table 1:** Patient laboratory test results with reference range

Test	Result	Reference Range
Complete Blood Count (CBC)		
Hemoglobin (Hb)	12.1 g/dL	Male: 13.8 - 17.2 g/dLFemale: 12.1 - 15.1 g/dL
White Blood Cells (WBC)	5100 cells/µL	4,500 - 11,000 cells/µL
Red Blood Cells (RBC)	5 million/µL	Male: 4.7 - 6.1 million/µLFemale: 4.2 - 5.4 million/µL
Hematocrit (Hct)	39.30%	Male: 40.7 - 50.3%Female: 36.1 - 44.3%
Platelets (PLT)	200,000 cells/µL	150,000 - 450,000 cells/µL
Mean Corpuscular Volume (MCV)	84 fL	80 - 100 fL
Mean Corpuscular Hemoglobin (MCH)	29 pg/cell	27 - 33 pg/cell
Mean Corpuscular Hemoglobin Concen(MCHC)	34 g/dL	32 - 36 g/dL
Liver Function Tests (LFT)		
Alanine Aminotransferase (ALT)	21 U/L	7 - 56 U/L
Aspartate Aminotransferase (AST)	15 U/L	10 - 40 U/L
Alkaline Phosphatase (ALP)	67 U/L	44 - 147 U/L
Total Bilirubin	1.0 mg/dL	0.1 - 1.2 mg/dL
Direct Bilirubin	0.2 mg/dL	0.0 - 0.3 mg/dL
Albumin	4.1 g/dL	3.5 - 5.0 g/dL
Total Protein	7.1 g/dL	6.0 - 8.3 g/dL
Gamma-Glutamyl Transferase (GGT)	21 U/L	9 - 48 U/L
Creatinine	0.9 mg/dL	0.6 - 1.2 mg/dL
Urea	12 mg/dL	7 - 20 mg/dL

Based on the above clinicopathological findings, a diagnosis of autoimmune progesterone dermatitis was made. The patient was reassured. She was asked to continue the same treatment that she had been using and put under periodic follow-up.

## Discussion

APD was first identified in 1921 when urticarial lesions were linked to the menstrual cycle [3,4]. Since then, fewer than 200 cases have been published in the literature [5,6].

APD is a rare type of hypersensitivity reaction that recurs monthly in women during the luteal phase of the menstrual cycle [2]. While the exact underlying mechanism remains unclear, the primary cause is believed to be a hypersensitivity to endogenous or exogenous progesterone, resulting in the clinical manifestations of the condition [3].

APD can manifest in various forms, including eczema, erythema multiforme, fixed drug eruptions, folliculitis, stomatitis, vesiculobullous lesions, urticaria, and anaphylaxis [1,7]. The trunk and limbs are the most commonly affected areas, but cases involving the face, oral mucosa, lips, and genitals have also been reported [6]. The lesions usually appear during the luteal phase of the menstrual cycle, starting 3 to 10 days before menstruation and resolving soon after [7-10]. A review of the literature is summarized in Table 2.

**Table 2:** Published cases of autoimmune progesterone dermatitis associated with the menstrual cycle N/A: Not Available

Author	Age (y)	Reaction	Duration	Relation to menstruation	Successful therapy
Banwaith et al. [2]	34	Widespread, pruritic, eczematous eruption	8 years	Appearing a few days before menses and resolving after menses	N/A
Prieto-Garcia et al. [8]	21	Fixed drug eruption	3 months	Beginning the day of her menses	Prednisone
Irshad et al. [3]	29	Painful blisters all over the body	1 year	Appearing 3-10 days prior to the menses and disappearing shortly after it	Topical clobetasol
Baptist et al. [9]	33	Urticaria and angioedema	21 years	Appearing 3-10 days prior to menses and ending 1-2 days after it	N/A
Özdemir et al. [11]	34	Urticaria	1 year	Appearing 6 days before menses and disappearing 1-2 days after it	Combined oral contraceptives
Our patient	50	Urticaria	3 years	Appearing a week before menses and ending after it	N/A

Diagnostic criteria for APD have been proposed by some authors. It includes: (a) skin lesions that appear with the start of menstruation, (b) a positive intradermal test, and (c) improvement after treatment with progesterone-inhibiting therapies [10].

The differential diagnosis includes urticarial vasculitis, acquired autoinflammatory syndromes, e.g., Schnitzler syndrome and adult-onset Still disease, hereditary autoinflammatory syndromes, e.g., FMF, MWS, and FCAS. The periodic nature of skin lesions, absence of systemic symptoms, such as fever, joint pain, neurological, gastrointestinal symptoms, absence of cold-induced urticarial skin lesions, absence of similar cases in the family, as well as the presence of normal skin biopsy findings in our patient ruled out these syndromes. In Schnitzler syndrome, the urticarial lesions are typically non-pruritic.

The primary treatment for APD focuses on ovulation suppression, with combined oral contraceptives, gonadotropin-releasing hormone (GnRH) agonists, danazol, and tamoxifen [3]. Our patient is suffering from APD while on OCP, so we do not know whether OCP in our patient is providing help or not. To confirm this, we need to do a challenge test to see whether she gets better or gets worse to confirm the therapeutic effect of OCP in our patient. Antihistamines and topical or systemic corticosteroids can be used to relieve cutaneous manifestations [9]. Our patient is under periodic follow-up. The skin lesions in our patient are temporarily improving on antihistamines and topical corticosteroids. For preventing the periodic recurrences of APD, the patient was referred to a gynecologist for the probable use of other anti-progesterone medications. In refractory cases, bilateral oophorectomy may be considered as a curative intervention [9].

Limitations

This case has several limitations. Definitive confirmation of APD was not achieved, as intradermal progesterone testing and progesterone challenge or withdrawal tests were not performed. Additionally, autoimmune and inflammatory laboratory tests were not obtained, which could have helped exclude alternative conditions. These limitations highlight the need for confirmatory hormonal and immunologic testing in future cases to strengthen diagnostic certainty.

## Conclusions

Autoimmune progesterone dermatitis (APD) is a rare autoimmune reaction to both endogenous and exogenous progesterone. Diagnosis is largely clinical, supported by intradermal progesterone testing and the patient’s response to antiprogesterone treatment. The differential diagnosis of periodic urticarial diseases are many; however, a high index of suspicion and thorough history and clinical examination are needed to make a diagnosis of APD whenever we have a case of periodic eczema, erythema multiforme, fixed drug eruptions, folliculitis, stomatitis, vesiculobullous lesions, urticaria, or anaphylaxis.
